# Chemical Corrosion-Water-Confining Pressure Coupling Damage Constitutive Model of Rock Based on the SMP Strength Criterion

**DOI:** 10.3390/ma16186234

**Published:** 2023-09-15

**Authors:** Youliang Chen, Huidong Tong, Qijian Chen, Xi Du, Suran Wang, Yungui Pan, Yang Dong, Hao Ma

**Affiliations:** 1 Department of Civil Engineering, School of Environment and Architecture, University of Shanghai for Science and Technology, Shanghai 200093, Chinapanyungui1997@163.com (Y.P.);; 2 Department of Engineering Geology and Hydrogeology, RWTH Aachen University, 52064 Aachen, Germany; 3 School of Civil and Environmental Engineering, University of New South Wales, Sydney, NSW 2052, Australia; 4 Department of Underground Architecture and Engineering, Tongji University, Shanghai 200093, China

**Keywords:** constitutive model, chemical corrosion, water damage, coupling effects, Weibull distribution, SMP strength criterion

## Abstract

Aiming at the problem of chemical-mechanics-hydro (C-M-H) action encountered by rocks in underground engineering, chemical damage variables, water damage variables, and force damage variables are introduced to define the degree of degradation of rock materials. Stone is selected as the sample for acid corrosion treatment at *pH* 3, 4, and 7, and a chemical damage factor is defined that coupled the *pH* value and duration of exposure. Then based on the spatial mobilized plane (SMP) criterion and the Lemaitre strain equivalence hypothesis, this research develops a constitutive model considering rock chemical corrosion-water-confining pressure damage. The proposed damage constitutive model employs the extremum method to ascertain the two Weibull distribution parameters (*m* and *F*_0_) by theoretical derivation and exhibits satisfactory conformity between the theoretical and experimental curves. The damage constitutive model can be consistent in the stress–strain characteristics of the rock triaxial compression process, which verifies the rationality and reliability of the model parameters. The model effectively represents the mechanical properties and damage characteristics of rocks when subjected to the combined influence of water chemistry and confinement. The presented model contributes to a better understanding of tangible rock-engineered structures subjected to chemical corrosion in underwater environments.

## 1. Introduction

The Sichuan–Tibet Railway is a crucial long-term initiative in China, bringing a substantial impact on the economic growth of the Southwest region. The Sichuan–Tibet Railway traverses the areas affected by acid rain and cold climate from the eastern to the western regions [1], characterized by intricate topography. Acid rain can significantly enhance the weathering of rock slopes, forming thick weathering layers by dissolving minerals and extending micro-cracks [2]. Rock joints are usually good channels for rainwater flow. Throughout the process of water migration, it can both alter and erode the adjacent medium through softening, modification, lubrication, and erosion while simultaneously undergoing dissolution, hydrolysis, and ion exchange with the rock. This can bring about changes in mineral composition, microstructure, and physical properties of the surrounding rocks, thus changing their macroscopic mechanical properties.

The integration of a constitutive model holds utmost importance in the framework of rock mechanics’ strength theory. Significant progress and achievements have been made in the theory of rock damage by researchers both domestically and internationally [3,4,5,6,7,8,9,10,11,12,13,14,15]. Rocks typically exhibit robust coupling properties as a result of the interplay between geological processes and intricate natural surroundings over extended periods. In certain rock engineering endeavors, intricate chemical and physical variables, such as acid precipitation and tectonic strain, frequently exert an influence on rocks situated in intricate geological settings with anomalous hydrogeological surroundings, occasionally resulting in harm such as pore formation. Such rocks exhibit alterations in their mechanical properties following exposure to tectonic stress, subsequently impinging upon the enduring stability of the project [16,17]. On a microscopic scale, rocks consist of interconnected mineral particles held together by bonding forces. However, these bonding forces can be dissolved by chemical solutions, leading to an accelerated process of rock damage. Simultaneously, the existence of tectonic stress induces the closure of micro-pores within rock particles, consequently influencing their mechanical strength [18,19]. A double-porosity constitutive model was proposed for geological materials subjected to uniaxial tension and compression. This model integrates the principles of elastic-plasticity and damage mechanics theories [20]. A linked model was put up by Ogata et al. [21] to explain how the *pH* affects the permeability of fractured rocks. The suggested model is used to predict the long-term permeability evolution of a naturally occurring granite barrier within a geological repository under underground circumstances. For stress-assisted corrosion, a brand-new mechano-chemical coupled peridynamic model is presented. According to the model, nonlocal dilatation of the material close to the corrosion front determines the activation-controlled anodic dissolution [22]. Sun et al. [23] defined the hydraulic chemistry-mechanics (HCM) coupling damage variable based on the law of damage mechanics and micro-strength distribution theory and established the constitutive equation for rock failure under the combination effect of hydration and cyclic water ingress. A time-dependent damage theory is presented by Borja et al. [24] for rocks that have undergone solid dissolution and mechanical deformation. The transition state theory, which posits that the rate of dissolution is a function of the reactive surface area assessed through the crack density in the volume, is the source of inspiration for the constitutive description. According to the experimental results [15], the duplication of chemical damage has a tremendous impact on the quality, wave velocity, porosity, and compression failure characteristics of the rock. To assess the solubility of anhydrite and gypsum in the aqueous phase, Taherdangkoo et al. [25] used feed-forward neural network (FFNN) and cascade-forward neural network (CFNN) models trained with a Bayesian regularization (BR) algorithm. Shi et al. [26] provided evidence for the effective depiction of rock deformation characteristics under freeze–thaw loading by the SMP criterion.

In summary, there have been significant advances in the field of rock constitutive modeling, but there are still limitations: (1) Insufficient research has been performed on the constitutive models of rock damage when conducting the bonding influence of chemical corrosion, water, and confining pressure. Furthermore, a significant portion of the extant literature on the strength criteria of rock microelements has opted to employ the maximum tensile strain criterion [27] and the Mohr–Coulomb criterion [28], primarily due to their straightforward mathematical formulations. The SMP criterion has been infrequently taken into account in current statistical damage constitutive models. Nonetheless, the aforementioned criteria failed to examine the direct impact of intermediate principal stress on rock reinforcement and disregarded the influence of intermediate principal stress. Therefore, expressing the stress–strain relationship accurately for materials resembling rock presents certain limitations. (2) The damage variables primarily comprise macroscopic mechanical performance indicators, including elastic modulus and wave velocity. Several studies have exhibited certain limitations due to the exclusion of chemical damage factors that take into account the *pH* value and duration. (3) The efficacy of strain-based damage constitutive models is limited due to the inadequacy of axial strain in accurately representing the strength of rock microelements [29]. Moreover, the assertion that the non-performance of trace elements adheres to the Drucker–Prager criterion lacks justification as the outcomes of the Drucker–Prager criterion are cautious [30]. The Mohr–Coulomb criterion is inadequate in characterizing the mechanical resistance of rock in regions of low stress or under tensile stress. Furthermore, the linear expression of its relationship is incongruous with the parabolic configuration of the rock failure envelope line [31]. (4) Traditional strength theory can explain some features of rock failure and damage based on microelement strength definition, but it is not sufficiently accurate to reflect specific stresses or strains. The SMP strength criterion has clear physical meanings for each variable while also overcoming the deficiency in the Mohr–Coulomb criterion, which does not take into account the effect of intermediate principal stress on rock strength, making it more suitable for rock-like materials than traditional criteria such as the M-C criterion and D-P criterion.

Additional investigation is necessary in light of the aforementioned issues. This article presents chemical damage variables incorporating *pH* value and time, water damage variables, and mechanical damage variables based on Weibull distribution. These variables aim to accurately reflect rock materials’ mechanical properties and deformation characteristics in acidic and water environments, building upon prior research. The mechanism of continuous damage mechanics is utilized. A constitutive model for rock chemical corrosion-water-confining pressure coupling damage was developed, utilizing the SMP criterion as the rock strength criterion and relying on the Lemaitre strain equivalence hypothesis. Subsequently, the model was subjected to validation through a comparative analysis with experimental curves, indicating a satisfactory conformity level. The present study elucidates the damage mechanism and damage mechanism of rocks that have undergone acid erosion under the influence of both water and confinement pressure.

## 2. Establishment of Rock Damage Constitutive Model under H-M-C Condition

### 2.1. Methodology

**Figure 1:**
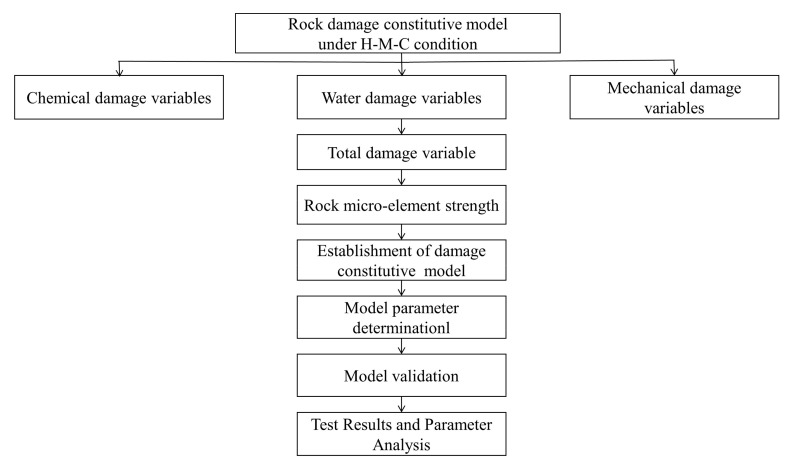
Technique flow chart for developing a rock damage constitutive model under H-M-C condition.

Figure 1 depicts the technique flow chart for developing a rock damage constitutive model under a chemical-mechanics-hydro condition.

### 2.2. Determination of Chemical Damage Variables

**Figure 2:**
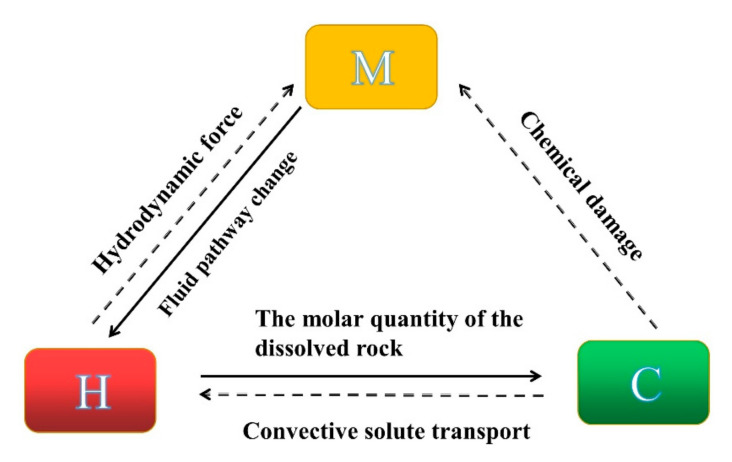
H-M-C coupled relation during rock hydrochemical damage and the fracturing process.

**Figure 3:**
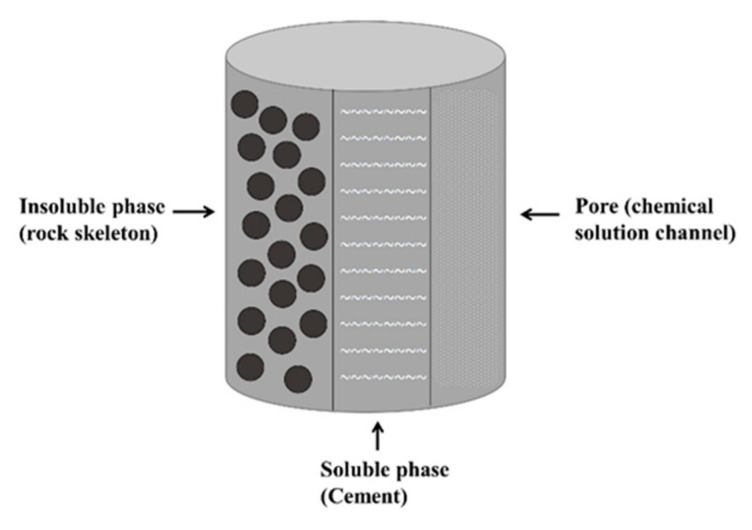
Hypothetical three-phase diagram of rock (acidic chemical environment).

The rock specimen used for mechanism analysis of the HMC coupled rock dissolution and fracturing processes is shown in Figure 2. The microstructure of rocks determines their mechanical characteristics. Due to diverse formation circumstances, rocks contain a variety of microstructures from a microscopic perspective, although the majority of them share the following traits: 1. Made up of mineral particles of irregular sizes; 2. Rife with pre-existing flaws such as microcracks and holes, as shown in Figure 3.

Chemical solutions seep into the rock through the interior pores of sandstones when they are exposed to a water–chemical environment. This occurs in the soluble mineral components and the solution of a specific ion chemical reaction [32,33], for example, in the acidic environment of calcium-cemented sandstone, where the cement will be constantly dissolved. On the one hand this can lead to sandstone internal microscopic defects that continue to increase, and on the other hand this can damage or weaken the connection state between the particles, which leads to the macro-mechanical properties occurring in varying degrees of deterioration.

**Figure 4:**
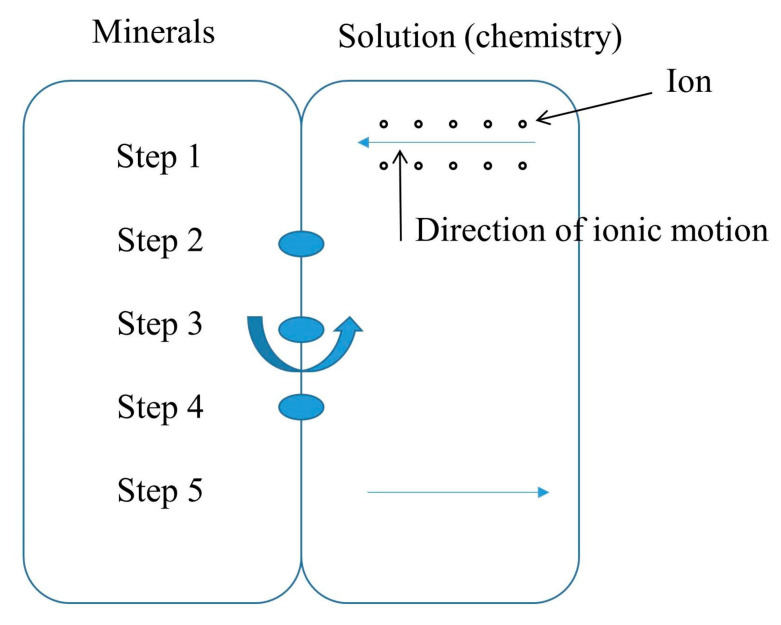
Stages in the process of rock hydrochemical reactions.

When sandstone is in an acidic chemical solution, a number of intricate physicochemical reactions take place that primarily result in the cement dissolving in the rock, changing the pore structure, weakening the intergranular connections, and degrading the mechanical properties. According to Mangold, D.C. [34] and other researchers’ findings, the water–rock reaction in a water–chemical solution can be broken down into the following five phases. Figure 4 illustrates the next five steps.

Step 1: ion solute migration from a chemical solution to the water–rock contact;

Step 2: at the water–rock contact, the initial interactions of ions with rock mineral components;

Step 3: chemical reaction at the water–rock interface between ions in the solution and soluble minerals;

Step 4: the product ions separate from the interface between water and rock;

Step 5: the ions generated by the chemical reaction diffuse and migrate out of the rock body.

In conclusion, the internal rock of calcareous cemented sandstone in an acidic chemical environment is the primary cause of mechanical damage. The chemical reaction between the cement and the hydrogen ions in the solution is the primary source of the mechanical damage since it results in cement loss through dissolving and weakens the bond between the particles. As time goes on, the cement is continuously dissolved. And because cement erodes over time, we can determine how much damage has been done to its mechanical characteristics and deformation characteristics by looking at how much of the soluble phase cement has been dissolved. Therefore, the degree of dissolving of the solubility can be used to analyze the degree of damage to its mechanical properties and deformation characteristics.

In practical applications, chemical corrosion may cause damage to the bonding materials and affect their load-bearing capacity, which is usually referred to as damage modulus. According to the study in the literature [35], the damage modulus caused by chemical corrosion can be calculated based on the effective load-bearing surface area of the bonding material.

(1) $$D_{C}=\frac{\Delta S^{d}}{{\left(S^{d}\right)}_{0}}={\left(\frac{\Delta r^{d}}{{\left(r^{d}\right)}_{0}}\right)}^{2}={\left(\frac{\Delta V^{d}}{{\left(V^{d}\right)}_{0}}\right)}^{\frac{2}{3}}={\left(\frac{\Delta \omega^{d}}{{\left(\omega^{d}\right)}_{0}}\right)}^{\frac{2}{3}}={\left(1-\frac{\omega^{d}}{{\left(\omega^{d}\right)}_{0}}\right)}^{\frac{2}{3}}$$

where ${\left(S^{d}\right)}_{0}$, ${\left(r^{d}\right)}_{0}$, and ${\left(V^{d}\right)}_{0}$ represent the effective load-bearing surface area, the radius of the load-bearing surface area, and the volume of the initial soluble bonding material of the sample, respectively; $\Delta S^{d}$, $\Delta r^{d}$, and $\Delta V^{d}$ represent the effective load-bearing surface area, the radius of the load-bearing surface area and the volume of the soluble bonding material during chemical corrosion, respectively; $\omega^{d}$ represents the number of moles of soluble material at any corrosion time, and ${\left(\omega^{d}\right)}_{0}$ represents the number of moles of the initial soluble material without corrosion.

According to the available experimental evidence [36], the primary cause of rock mineral composition degradation in a hydrochemical setting is the interaction between the soluble bonding materials present in the rock and acid or alkali, as well as the direct reaction between these materials and water. This paper will center its attention on the chemical corrosion process of rocks when exposed to acidic conditions. The primary mechanism responsible for the loss of mass is the chemical reaction between the soluble bonding material present in the rock and the acid and water. Upon exposure to acid, the cations present in the bonding material undergo a chemical reaction, leading to their dissolution and consequent loss of the bonding material. Furthermore, the aqueous medium has the potential to solubilize additional constituents present in the soluble bonding material when subjected to acidic conditions, thereby intensifying the deterioration of the bonding material.

The substances in the mineral composition of the rock react with the acid with the general chemical equation:

(2) $$\;m_{i}A_{i}+n_{i}H^{+}\to p_{i}B_{i}+q_{i}C_{i\;}$$

where *m_i_*, *n_i_*, *p_i_*, and *q_i_* are the balancing coefficients of the chemical equation, *A_i_* and *H^+^* are reactants, and *B_i_* and *C_i_* are products.

Based on the relationship between *pH* and *H^+^* concentration,

(3) $$pH=-\mathrm{lg}c\;(H^{+})$$

The determination of the number of soluble substances lost in a chemical reaction can be achieved by analyzing the alterations in H^+^ concentration before and following an acid-base reaction, as well as the correlation between the chemical Equation (2) and the reactant composition, without taking into account the sequence of chemical reactions. The principle that the quantity of protons in a chemical reaction is equivalent to the charge can be applied to transform the mass proportion of reactants into the mass proportion of H^+^ to determine the quantity of soluble substances in moles.

(4) $$\Delta \omega^{d}=\sum_{i=1}^{M}\frac{m_{i}\alpha_{i}}{n_{i}}(c_{H^{+}}(t)-c^{0}{}_{H^{+}})V_{CS}$$

In the equation, $m_{i}$ and $n_{i}$ are balancing coefficients, $c_{H^{+}}(t)$ and $c^{0}{}_{H^{+}}$ are ion concentrations at time *t* and initial time, respectively, $\alpha_{i}$ represents the initial mole fraction of the reactant A_i_ in all reactants that can react with *H^+^*, $V_{CS}$ representing the volume of the chemical solution.

Combining Equations (1), (3) and (4), the chemical damage factor considering the initial *pH* value and time t can be obtained, and its expression is as follows:

(5) $$D_{c}=1-{\left|\frac{\sum_{i=1}^{M}\frac{m_{i}\alpha_{i}}{n_{i}}(10^{-pH_{0}}-10^{-pH(t)})V_{CS}}{{\left(\omega^{d}\right)}_{0}}\right|}^{\frac{2}{3}}$$

### 2.3. Determination of Water Damage Variables

Water has the potential to engage in a chemical reaction with N number of related substances *D*_i_ present in rocks when it is in a solution. The underlying assumption of the reaction is that water is abundant, thereby facilitating a rapid completion of the reaction and rendering the impact of time negligible. During the progression of the chemical reaction, a specific quantity of soluble compounds will undergo dissolution and subsequently react with water, leading to the formation of a sequence of ions and molecules that will manifest within the resultant solution. Thus, the molar quantity of the dissolved concretion that has been lost can be determined by computing the molar quantities of both the reactants and the products involved.

(6) $$D_{w}=1-\frac{\sum_{i=1}^{N}\frac{m_{D_{i}}}{M_{i}}}{{\left(\omega^{d}\right)}_{0}}$$

where $m_{Di}$ and *M*_i_ represent the mass of the dissolved concretion *D_i_* and the molar mass of *D_i_*, respectively.

### 2.4. Determination of Mechanical Damage Variables

The presence of a significant number of randomly distributed pores within rocks is attributed to their heterogeneity. Consequently, when subjected to external loads, the deterioration mechanism of rocks typically encompasses the initiation, progression, and aggregation of voids, which exhibit a strong correlation with the number of impaired microconstituents. In general, the deficiency of microelements in rocks exhibits a degree of stochasticity.

To better describe the damage changes of rocks at different stress levels, this paper defines the damage variable $D_{Q}$. This variable compares the number of microelements that have been damaged, n, with the total number of microelements *N* in the initial state, reflecting the degree of damage of rocks under the stress level *Q*.

(7) $$D_{Q}=\frac{n}{N}$$

Under external loads, the failure of rock microelements usually exhibits randomness. The Krajcinovic model shows that the damage variable of rocks can be defined as the probability (*P*) of microelement failure. If the probability density function of microelement failure is represented as ϕ(x), then *P* is the cumulative distribution function of *F*:

(8) $$D_{Q}=P=\int_{0}^{F}\phi (x)\mathrm{dx}$$

Research has indicated that in the progress of a statistical constitutive model for rock damage, the adoption of a Weibull or normal distribution to represent the strength of rock microelements can yield more favorable outcomes in the investigation of the damage evolution characteristics that occur during rock failure. In contrast to the normal distribution, the Weibull distribution is a less restrictive distribution category that is more representative and broadly relevant to the distribution of stochastic variables. Therefore, this paper assumes that the strength of rock microelements follows a Weibull distribution [37]:

(9) $$\begin{array}{l} \phi (F)=\left\{\begin{array}{ll} \frac{m}{F_{0}}{\left(\frac{F}{F_{0}}\right)}^{m-1}exp[-{\left(\frac{F}{F_{0}}\right)}^{m}] & F>0\\ 0 & F\leq 0 \end{array}\right. \end{array}$$

where *m* and $F_{0}$ are the statistical parameters of the Weibull distribution, which reflects the degree of dispersion of the rock damage distribution; *F* represents the strength of microelements.

Based on Equation (8), the damage variable of rocks under loading can be obtained as follows:

(10) $$D_{Q}=\int_{0}^{F}\phi (x)\mathrm{dx}=1-\exp[-{\left(\frac{F}{F_{0}}\right)}^{m}]$$

### 2.5. Determination of Total Damage Variable of Rock under H-M-C Condition

The combined effects of chemical corrosion and loading result in varied damage characteristics in rocks. Therefore, in cases where chemical corrosion, water damage, and confining pressure coexist, the aggregate extent of rock deterioration cannot be regarded as a mere summation of the individual damages. In this study, it is assumed that the undamaged rock, the rock that has undergone chemical corrosion damage, and the rock that has undergone chemical corrosion–load coupling damage correspond to effective volumes *V*_0_, *V*_1_, and *V*_2_, respectively. The effective volume of rock under chemical corrosion–water damage and force coupling is *V*_3_. The ratio of defect volume to total volume is used to represent damage, that is, the ratio of defect volume to total volume.

(11) $$D_{C}=1-\frac{V_{1}}{V_{0}}$$

(12) $$D_{Q}=1-\frac{V_{2}}{V_{1}}$$

(13) $$D_{W}=1-\frac{V_{3}}{V_{2}}$$

The overall damage variable of rocks under the coupling effects of chemical corrosion, water, and confining pressure can be determined by combining Equations (11) and (12):

(14) $$D_{S}=1-\frac{V_{3}}{V_{0}}=D_{Q}+D_{C}+D_{W}-D_{Q}D_{C}-D_{C}D_{W}-D_{Q}D_{W}+D_{C}D_{Q}D_{W}$$

where $D_{S}$ represents the total damage variable of rocks under the coupling effects of chemical corrosion, water, and confining pressure.

By substituting Equations (5), (6) and (10) into Equation (14), the overall damage variable of rocks can be determined under the combined influence of chemical corrosion, water, and confining pressure.

(15) $$D_{S}=1-[1-{\left|\frac{\sum_{i=1}^{M}\frac{m_{i}\alpha_{i}}{n_{i}}(10^{-pH_{0}}-10^{-pH(t)})V_{CS}}{{\left(\omega^{d}\right)}_{0}}\right|}^{\frac{2}{3}}-\left(\frac{\sum_{i=1}^{N}\frac{m_{Di}}{M_{i}}}{{\left(\omega^{d}\right)}_{0}}\right)+\left(\frac{\sum_{i=1}^{N}\frac{m_{Di}}{M_{i}}}{{\left(\omega^{d}\right)}_{0}}\right){\left|\frac{\sum_{i=1}^{M}\frac{m_{i}\alpha_{i}}{n_{i}}(10^{-pH_{0}}-10^{-pH(t)})V_{CS}}{{\left(\omega^{d}\right)}_{0}}\right|}^{\frac{2}{3}}]\exp[-{\left(\frac{F}{F_{0}}\right)}^{m}]$$

### 2.6. Determination of Rock Micro-Element Strength under Confining Pressure

The SMP criterion proposed by Matsuoka and Nakai [38] in 1974, which considers the effect of the medium principal stresses, establishes a strength equation for soils under three-dimensional conditions, and this damage criterion assumes that the material breaks down when the ratio of the shear stresses to the positive stresses reaches a certain value. The SMP criterion has a stronger physical derivation process, and the physical significance of each variable is clear. At the same time, it overcomes the defects of the Mohr–Coulomb criterion that does not consider the effect of the intermediate principal stresses on the strength of the rock and its applicability to certain rock-like materials is more satisfactory. The expression is:

(16) $$\frac{I_{1}I_{2}}{I_{3}}=K_{1}=8\tan^{2}\varphi +9$$

where *I*_1_, *I*_2_, and *I*_3_ are the first invariant of the stress tensor, the second invariant of the stress tensor, and the third invariant of the stress tensor, *φ* is the angle of internal friction of the rock, and *K*_1_ is the material constant.

The above formulation of the SMP criterion is only applicable to frictional materials but not to c-*φ* materials. Matsuoka et al. [39] extended the criterion to accommodate both non-cohesive and cohesive materials and proposed the cohesive stress $\sigma_{0}$, which is expressed as:

(17) $$\sigma_{0}=c\cot\varphi$$

The modified SMP guideline expression is:

(18) $$\frac{{\overset{⌢}{I}}_{1}{\overset{⌢}{I}}_{2}}{{\overset{⌢}{I}}_{3}}=K_{1}=8\tan^{2}\varphi +9$$

where

(19) $$\begin{array}{cc} {\hat{I}}_{1}= & \sigma_{1}^{*}+\sigma_{2}^{*}+\sigma_{3}^{*}+3\sigma_{0}\\ {\hat{I}}_{2}= & \left(\sigma_{1}^{*}+\sigma_{0}\right)\left(\sigma_{2}^{*}+\sigma_{0}\right)+\left(\sigma_{2}^{*}+\sigma_{0}\right)\left(\sigma_{3}^{*}+\sigma_{0}\right)\\ & +\left(\sigma_{3}^{*}+\sigma_{0}\right)\left(\sigma_{1}^{*}+\sigma_{0}\right)\\ {\hat{I}}_{3}= & \left(\sigma_{1}^{*}+\sigma_{0}\right)\left(\sigma_{2}^{*}+\sigma_{0}\right)\left(\sigma_{3}^{*}+\sigma_{0}\right) \end{array}$$

By combining Equations (18) and (19) and based on the associated flow rule, the expression for the SMP criterion of viscous materials can be obtained as:

(20) $$\begin{array}{c} \frac{\sigma_{1}^{*}+\sigma_{0}}{\sigma_{3}^{*}+\sigma_{0}}=A=\frac{1}{4}\left(\sqrt{8\tan^{2}\varphi +9}+\right.\\ {\left.\sqrt{8{\mathrm{san}}^{2}\varphi +6-2\sqrt{8\tan^{2}\varphi +9}}-1\right)}^{2} \end{array}$$

Based on Equation (20), the SMP criterion-based microelement strength *F* is obtained as:

(21) $$F=f\;(\sigma_{1},\;\sigma_{2},\;\sigma_{3})=\frac{\sigma_{1}{}^{*}+\sigma_{0}}{\sigma_{3}{}^{*}+\sigma_{0}}$$

According to the generalized Hooke’s law and the principle of train equivalence, $\;\sigma_{2}$ = $\sigma_{3}$, we have:

(22) $$\left\{\begin{array}{l} \varepsilon_{1}=\frac{\sigma_{1}{}^{*}-2\nu \sigma_{3}{}^{*}}{E}\\ \sigma_{1}{}^{*}=\frac{\sigma_{1}}{1-D_{Q}}\\ \sigma_{3}{}^{*}=\frac{\sigma_{3}}{1-D_{Q}}\\ 1-D_{Q}=\frac{\left(\sigma_{1}-2\nu \sigma_{3}\right)}{E\varepsilon_{1}} \end{array}\right.$$

By combining Equation (22) and substituting effective stress with nominal stress in Equation (21), we can obtain the elemental strength *F* of rocks under confining pressure based on the SMP criterion, that is:

(23) $$F=\frac{E\varepsilon_{1}+2\nu \sigma_{3}+\sigma_{0}}{\sigma_{3}+\sigma_{0}}$$

### 2.7. Rock Damage Constitutive Model under H-M-C Condition

By substituting Equations (5), (6) and (23) into Equation (15), the damage evolution equation of rocks under the coupling effects of chemical corrosion and load can be obtained, which is:

(24) $$D_{s}=[1-[1-{\left|\frac{\sum_{i=1}^{M}\frac{m_{i}\alpha_{i}}{n_{i}}\left(10^{-pH_{0}}-10^{-pH\left(t\right)}\right)V_{CS}}{{\left(\omega^{d}\right)}_{0}}\right|}^{\frac{2}{3}}-\left(\frac{\sum_{i=1}^{N}\frac{m_{Di}}{M_{i}}}{{\left(\omega^{d}\right)}_{0}}\right)\;\;+\left(\frac{\sum_{i=1}^{N}\frac{m_{Di}}{M_{i}}}{{\left(\omega^{d}\right)}_{0}}\right){\left|\frac{\sum_{i=1}^{M}\frac{m_{i}\alpha_{i}}{n_{i}}\left(10^{-pH_{0}}-10^{-pH\left(t\right)}\right)V_{CS}}{{\left(\omega^{d}\right)}_{0}}\right|}^{\frac{2}{3}}]\exp[-\left(\frac{E\varepsilon_{1}+2\mu \sigma_{3}+\sigma_{0}}{\left(\sigma_{3}+\sigma_{0}\right)F_{0}}{)}^{m}\right]$$

According to Lemaitre’s strain equivalence principle and the concept of effective stress, the strain observed in a damaged rock under nominal stress is equivalent to the effective strain exhibited by an undamaged rock under effective stress (where nominal stress refers to the measured stress during experimentation). The establishment of the constitutive relationship of rock damage can be achieved through the following means:

(25) $$\sigma_{i}^{*}=\frac{\sigma_{i}}{1-D_{S}}(i=1,2,3)$$

where $\sigma_{i}^{*}$ is the effective stress in the rock; $\sigma_{i}$ is the nominal stress in the rock and $D_{S}$ is the damage variable in the rock. Then, according to the generalized Hooke’s law, it follows that

(26) $$\varepsilon_{i}^{*}=\frac{\sigma_{1}{}^{*}-\nu (\sigma_{2}^{*}+\sigma_{3}^{*})}{E_{n}}$$

where *E*_n_ is the modulus of elasticity of the rock at *pH* n; *v* is the Poisson’s ratio and $\varepsilon_{i}^{*}$ is the effective strain corresponding to the effective stress $\sigma_{i}^{*}$.

According to the deformation coordination condition, it is obtained that

(27) $$\varepsilon_{i}^{*}=\varepsilon_{i}$$

From Equations (25)–(27), a rock damage constitutive relationship is obtained

(28) $$\sigma_{1}=E_{n}\varepsilon_{1}(1-D_{s})+\nu (\sigma_{2}+\sigma_{3})$$

In accordance with the experimental principle of the rock triaxial compression test, the confining pressure load is first applied during the test, and then the axial load is applied after the confining pressure stabilizes to the predetermined value. Therefore, before the axial load is applied, the rock will experience initial axial strain under the action of confining pressure. The recorded axial strain is the difference between the axial stress and the confining pressure $\sigma_{3}$, that is, the axial stress minus the confining pressure.

(29) $$\sigma_{1t}=\sigma_{1}-\sigma_{3}$$

The initial axial strain of rock under the action of confining pressure is:

(30) $$\varepsilon_{10}=\frac{1-2v}{E_{T}}\sigma_{3}$$

Since the test results do not include the initial axial strain generated before the axial pressure is applied, the actual axial strain $\varepsilon_{1}$ is the sum of the measured axial strain $\varepsilon_{1t}$ during the experiment and the initial axial strain $\varepsilon_{10}$, which is:

(31) $$\varepsilon_{1}=\varepsilon_{1t}+\varepsilon_{10}$$

The rock chemical corrosion-water-confining pressure coupling damage constitutive model can be obtained by substituting Equations (24) and (29)–(31) into Equation (28).

(32) $$\sigma_{1t}=[E_{n}\varepsilon_{1t}+(1-2\nu )\sigma_{3}][1-{\left|\frac{A}{{\left(\omega^{d}\right)}_{0}}\right|}^{\frac{2}{3}}-\left(\frac{\sum_{i=1}^{N}\frac{m_{Di}}{M_{i}}}{{\left(\omega^{d}\right)}_{0}}\right)+\left(\frac{\sum_{i=1}^{N}\frac{m_{Di}}{M_{i}}}{{\left(\omega^{d}\right)}_{0}}\right){\left|\frac{A}{{\left(\omega^{d}\right)}_{0}}\right|}^{\frac{2}{3}}]\exp[-{\left(\frac{F}{F_{0}}\right)}^{m}]+(2\nu -1)\sigma_{3}$$

where

(33) $$A=\sum_{i=1}^{M}\frac{m_{i}\alpha_{i}}{n_{i}}(10^{-pH_{0}}-10^{-pH(t)})V_{CS}$$

(34) $$F=\frac{E\varepsilon_{1}+2\nu \sigma_{3}+\sigma_{0}}{\sigma_{3}+\sigma_{0}}$$

## 3. Model Parameter Determination

The present study employs the extremum method to ascertain the two Weibull distribution parameters, namely *m*, and *F*_0_, that are necessary for the rock damage constitutive model proposed herein. The said parameters are determined based on the peak point ($\varepsilon_{p}$, $\sigma_{p}$) of the rock stress–strain curve. The specific solution process is as follows.

The peak stress $\sigma_{p}$ and the corresponding peak strain $\varepsilon_{p}$ satisfy the following two geometrical conditions:

(35) $$\varepsilon =\varepsilon_{p},\sigma_{1}=\sigma_{p}$$

(36) $$\varepsilon =\varepsilon_{p},\frac{d\sigma_{1}}{d\varepsilon_{1}}=0$$

First, substituting Equation (35) with Equation (32) yields

(37) $$[1-{\left|\frac{A}{{\left(\omega^{d}\right)}_{0}}\right|}^{\frac{2}{3}}-\left(\frac{\sum_{i=1}^{N}\frac{m_{Di}}{M_{i}}}{{\left(\omega^{d}\right)}_{0}}\right)+\left(\frac{\sum_{i=1}^{N}\frac{m_{Di}}{M_{i}}}{{\left(\omega^{d}\right)}_{0}}\right){\left|\frac{A}{{\left(\omega^{d}\right)}_{0}}\right|}^{\frac{2}{3}}]\exp[-{\left(\frac{F_{SC}}{F_{0}}\right)}^{m}]=\frac{\sigma_{p}+(1-2v)\sigma_{3}}{E\varepsilon_{p}+(1-2\nu )\sigma_{3}}$$

where

(38) $$A=\sum_{i=1}^{M}\frac{m_{i}\alpha_{i}}{n_{i}}(10^{-pH_{0}}-10^{-pH(t)})V_{CS}$$

(39) $$F_{SC}=\frac{E\varepsilon_{1}{}_{t}+2\nu \sigma_{3}+\sigma_{0}}{\sigma_{3}+\sigma_{0}}$$

*F_SC_* in Equation (39) is the *F* corresponding to the extreme value point of the stress–strain curve.

Taking the partial derivative of Equation (32), we obtain:

(40) $$\frac{\partial \sigma_{1t}}{\partial \varepsilon_{1t}}\left|{}_{\begin{array}{l} \sigma_{1t}=\sigma_{p}\\ \varepsilon_{1t}=\varepsilon_{p} \end{array}}\right.={\left|\frac{A}{{\left(\omega^{d}\right)}_{0}}\right|}^{\frac{2}{3}}\left(\frac{\sum_{i=1}^{N}\frac{m_{Di}}{M_{i}}}{{\left(\omega^{d}\right)}_{0}}\right)\exp[-{\left(\frac{F}{F_{0}}\right)}^{m}]\cdot \{E_{n}-\frac{m[E_{n}\varepsilon_{p}+(1-2v)\sigma_{3}]}{F_{sc}}{\left(\frac{F_{SC}}{F_{0}}\right)}^{m}\frac{\partial F_{SC}}{\partial \varepsilon_{1t}}\}$$

From Equation (36), it is known that:

(41) $$E_{n}-\frac{m[E_{n}\varepsilon_{p}+(1-2v)\sigma_{3}]}{F_{sc}}{\left(\frac{F_{SC}}{F_{0}}\right)}^{m}\frac{\partial F_{SC}}{\partial \varepsilon_{1t}{}_{}}=0$$

Taking the partial derivative of Equation (39), we obtain:

(42) $$\frac{\partial F_{sc}}{\partial \varepsilon_{1t}}\left|{}_{\begin{array}{l} \sigma_{1t}=\sigma_{p}\\ \varepsilon_{1t}=\varepsilon_{p} \end{array}}\right.=\frac{E_{n}}{\sigma_{3}+\sigma_{0}}$$

Substituting Equation (42) into Equation (41), we obtain:

(43) $$\begin{array}{l} {\left(\frac{F_{sc}}{F_{0}}\right)}^{m}=\frac{F_{\mathrm{sc}}(\sigma_{3}+\sigma_{0})}{m[E_{n}\varepsilon_{p}+(1-2v)\sigma_{3}]}=\frac{E_{n}\varepsilon_{p}+\sigma_{3}+\sigma_{0}}{m[E_{n}\varepsilon_{p}+(1-2v)\sigma_{3}]} \end{array}$$

Combining Equation (37), we can obtain expressions for the parameters *m* and *F*_0_:

(44) $$m=\frac{1}{\ln[D_{CW}(\frac{E_{n}\varepsilon_{p}+(1-2\nu )\sigma_{3}}{\sigma_{p}+(1-2v)\sigma_{3}})]}\frac{E_{n}\varepsilon_{p}+\sigma_{3}+\sigma_{0}}{\left[E_{n}\varepsilon_{p}+(1-2v)\sigma_{3}\right]}$$

where

(45) $$D_{CW}=1-{\left|\frac{A}{{\left(\omega^{d}\right)}_{0}}\right|}^{\frac{2}{3}}-\left(\frac{\sum_{i=1}^{N}\frac{m_{Di}}{M_{i}}}{{\left(\omega^{d}\right)}_{0}}\right)+\left(\frac{\sum_{i=1}^{N}\frac{m_{Di}}{M_{i}}}{{\left(\omega^{d}\right)}_{0}}\right){\left|\frac{A}{{\left(\omega^{d}\right)}_{0}}\right|}^{\frac{2}{3}}$$

(46) $$F_{0}=\frac{E_{n}\varepsilon_{p}+\sigma_{3}+\sigma_{0}}{\left(\sigma_{3}+\sigma_{0}\right)}{\left|\frac{\left(E_{n}\varepsilon_{p}+(1-2v)\sigma_{3}\right)}{E_{n}\varepsilon_{p}+\sigma_{3}+\sigma_{0}}m\right|}^{\frac{{}^{1}}{m}}$$

By using the parameter determination method described above and substituting the obtained parameters *m* and *F*_0_ into Equation (32), we can obtain the damage constitutive model of rock under the coupling effect of chemical corrosion-water-confining pressure.

## 4. The Validation of Rock Damage Constitutive Model under H-M-C Condition

### Parameter Identification

The fundamental aspects of rock damage resulting from acidic solution corrosion are characterized by the degradation of the rock’s particle framework and the concomitant augmentation of its porosity. To ascertain the rationality and precision of the model, the present study references the findings of Wang [40]. Blocks of sandstone were gathered from a Chinese hydroelectric plant site. At a macroscopic level, the blocks are quite homogeneous. The sandstone under study is deeply red because it is rich in oxides, primarily iron oxide. Wang conducted empirical investigations to quantify the deviatoric stress, axial strain, and radial strain of rocks after undergoing corrosion by acidic solutions with varying *pH* levels. The solutions were characterized by *pH* values of 3, 4, and 7, while a control group remained untreated. Table 1 displays the mineral composition of sandstone subjected to corrosion by solutions of varying *pH.*

The chemical damage factor *D_c_* can be obtained by combining the mineral composition and Equation (5) of the sandstone.

The results of the chemical composition analysis of sandstone (see Table 1) show that the chemical composition of sandstone is mainly composed of SiO_2_, Al_2_O_3_, and Fe_2_O_3_. The maximum and minimum values of the same component differ significantly, and their average was taken. Among them, the SiO_2_ content of sandstone was the highest with an average value of 51.2%; the average content of Al_2_O_3_ is 4.6%; the average content of Fe_2_O_3_ is 4.6%; the average content of MgO is 3.5%; the total content of the unstable components Na_2_O, K_2_O, and CaO is 3.4–15.0%, although the content is small but active in nature.

In Table 1, SiO_2_ is insoluble in water and difficult to react with acidic solutions. Existing data have shown that the dissolution of KAlSi_3_O_8_ and NaAlSi_3_O_8_ in feldspar at room temperature in acidic solutions is very small and can be ignored. Therefore, feldspar is considered an insoluble matrix. The soluble cementing materials that react with acids are mainly Fe_2_O_3_, MgO, and Al_2_O_3_ in the sandstone. The chemical reaction equation between clay minerals in sandstone and acidic solutions mainly includes:

(1) Sandstone reacts with acid in the following main ways:

(47) $$\begin{array}{l} {\mathrm{NaAlSi}}_{3}O_{8}+4H^{+}+4H_{2}O\to 3H_{4}{\mathrm{SiO}}_{4}+{\mathrm{Na}}^{+}+{\mathrm{Al}}^{3+}\\ {\mathrm{KAlSi}}_{3}O_{8}+4H^{+}+4H_{2}O\to 3H_{4}{\mathrm{SiO}}_{4}+K^{+}+{\mathrm{Al}}^{3+}\\ {\mathrm{CaAl}}_{2}{\mathrm{Si}}_{2}O_{8}+8H^{+}\to {\mathrm{Ca}}^{2+}+2{\mathrm{Al}}^{3+}+2H_{4}{\mathrm{SiO}}_{4}\\ {\mathrm{KAl}}_{3}{\mathrm{Si}}_{3}O_{10}{\left(\mathrm{OH}\right)}_{2}+10H^{+}\to 3H_{4}{\mathrm{SiO}}_{4}+K^{+}+3{\mathrm{Al}}^{3+}\\ {\mathrm{CaCO}}_{3}+2H^{+}\to {\mathrm{Ca}}^{+}+H_{2}O+{\mathrm{CO}}_{2}↑\\ \mathrm{MgO}+2H^{+}\to {\mathrm{Mg}}^{2+}{+H}_{2}O\\ {\mathrm{Al}}_{2}O_{3}{+6H}^{+}\to {2\mathrm{Al}}^{3+}{+3H}_{2}O\\ {\mathrm{Fe}}_{2}O_{3}{+6H}^{+}\to {2\mathrm{Fe}}^{3+}{+3H}_{2}O\\ {\mathrm{CaO}+2H}^{+}\to {\mathrm{Ca}}^{2+}+H_{2}O\\ {\mathrm{Na}}_{2}O+2H^{+}\to 2{\mathrm{Na}}^{+}+H_{2}O \end{array}\}$$

(2) Sandstone reacts with water in the following main way:

(48) $$\left.\begin{array}{l} SiO_{2}+2H_{2}O\to H_{4}SiO_{4}\\ K_{2}O+H_{2}O\to 2K^{+}+2OH^{-}\\ Na_{2}O+H_{2}O\to 2Na^{+}+OH^{-}\\ C{\mathrm{aO}+H}_{2}O\to {\mathrm{Ca}(\mathrm{OH})}_{2}\\ {\mathrm{MgO}+H}_{2}O\to {\mathrm{Mg}(\mathrm{OH})}_{2} \end{array}\right\}$$

It is clear from the aforementioned reactions that the sandstone minerals were chemically interacted with water and acidic solutions to form Ca^2+^, Na^+^, Mg^2+^, K^+^, and other cations. Under the influence of acid corrosion, the interior mineral components of sandstone dissolve and precipitate as ions.

The sandstone minerals’ calcite and calcium feldspar are incredibly fragile in the presence of acid, which increases the rate of Ca^2+^ dissolution. Since sodium feldspar predominates in the mineral composition of the rock sample and the dissolution rate of Ca^2+^ increases rapidly in the early stages of immersion, Ca^2+^ has the biggest dissolving rate of the four cations, followed by Na+. Of the four cations, Ca^2+^ dissolves the fastest, followed by Na^+^, and K^+^ and Mg^2+^ are primarily found in clay minerals such illite, muscovite, and montmorillonite. Illite, muscovite, montmorillonite, and other clay minerals are the principal sources of K^+^ and Mg^2+^ due to the low amount of K^+^ and Mg^2+^ produced and precipitated after acid exposure.

The fundamental premise of the reaction is that water is abundant, allowing for a quick completion of the reaction and insignificant effects of time. A certain number of soluble compounds will dissolve as the chemical reaction progresses, and when they do, they will react with water to generate a series of ions and molecules that will appear in the solution that results. Thus, by calculating the molar quantities of both the reactants and the products involved, it is possible to establish the molar quantity of the dissolved concretion that has been lost.

The *pH* level of the immersion solution has a direct impact on how much each cation dissolves. The chemical reaction between H^+^ and the minerals in the rock samples is stronger at lower *pH* values, and thus increases the pace at which the ions contained in the minerals dissolve. The chemical interaction between calcite (CaCO_3_) and strong acids is fairly simple to carry out and results in the formation of Ca^2+^ compounds, which then dissolve in the solution. The maximum rate of Ca^2+^ dissolution occurs in the solution; in an acidic environment, clay minerals such as montmorillonite, illite, and others dissolve, hydrolyze, and exchange ions. Acidic environments cause clay minerals such as montmorillonite, illite, and others to dissolve, hydrolyze, and exchange ions.

Based on the mineral composition of sandstone in Table 1, the total moles of soluble cementing materials in the sandstone used in the experiment can be calculated as 1.9876 mol. As shown in Table 2, in the process of calculating the chemical damage factor *D*_c_ using Equation (5), the soaking time *t* in Equation (5) should be selected as 70 h when the *pH* reaches preliminary stability rather than the entire soaking time of 200 h. After statistics, the number of moles of soluble cementing materials damaged by the red sandstone soaked in 1 L of chemical solution with an initial *pH* of 3 and reacted with H^+^ was found to be 3.8280 × 10^−4^ mol, and *D*_c(PH = 3)_ = $3.3017\times 10^{-2}$; the number of moles of soluble cementing materials damaged by the red sandstone soaked in 1 L of chemical solution with an initial *pH* of 4 and reacted with H^+^ was $3.8032\times 10^{-5}$ mol, and *D*_c(PH = 4)_ = $7.1536\times 10^{-4}$; the number of moles of soluble cementing materials damaged by the red sandstone soaked in 1 L of chemical solution with an initial *pH* of 7 and reacted with H^+^ was 0, and the number of moles of soluble cementing materials damaged by reacting with water was 0.625 mol, and *D*_w_ = 0.3144.

Substituting the data from Table 2 and the experimentally measured data into Equations (44) and (45), we obtainws the Weibull distribution parameters *m* and *F*_0_ under different *pH* values and confining pressures, as shown in Table 3.

## 5. Test Results and Parameter Analysis

**Figure 5:**
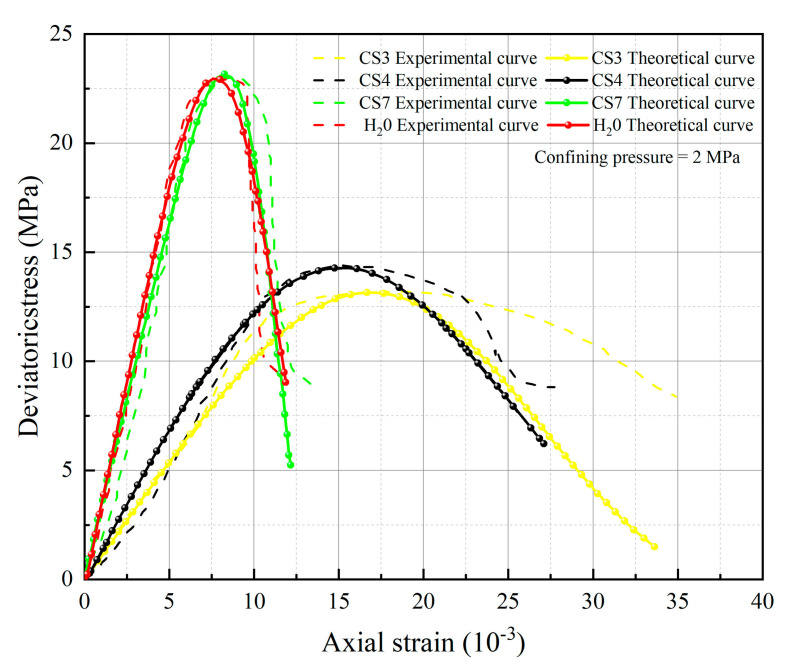
Comparison of theoretical and experimental stress–strain curves for an enclosing pressure of 2 MPa.

**Figure 6:**
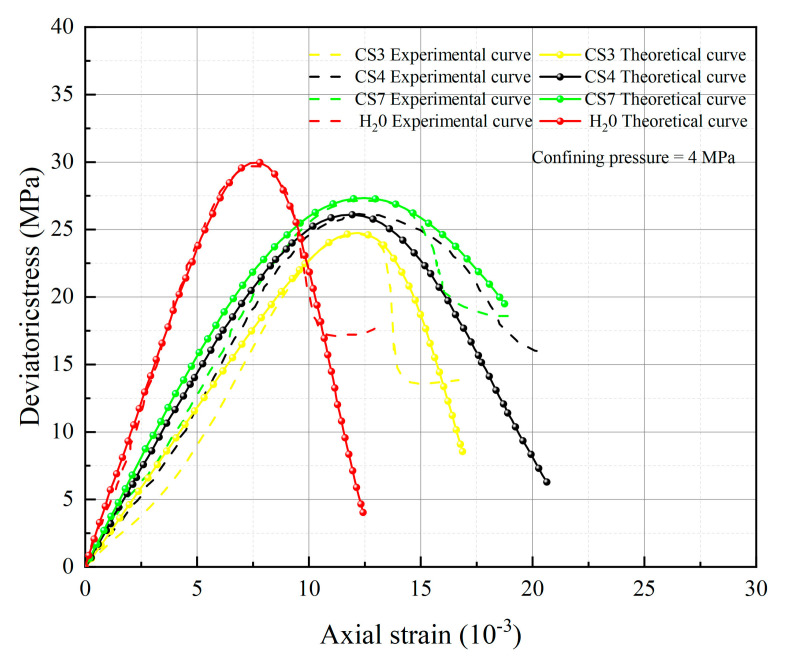
Comparison of theoretical and experimental stress–strain curves for an enclosing pressure of 4 MPa.

**Figure 7:**
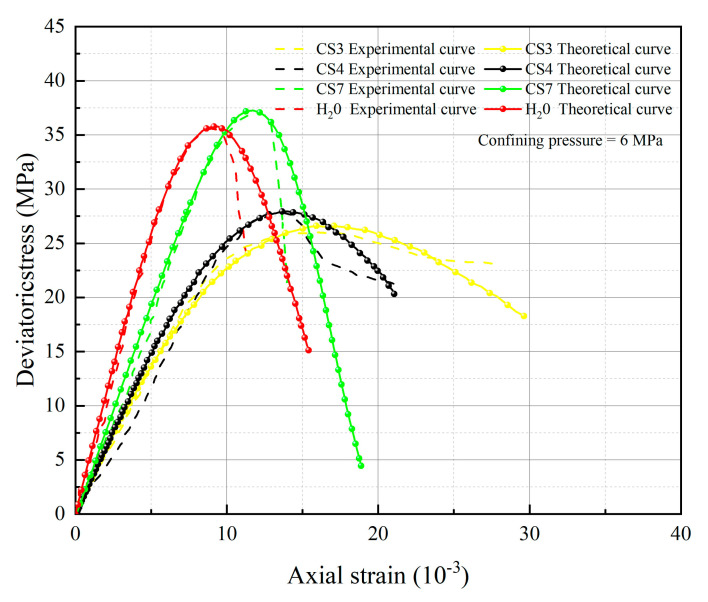
Comparison of theoretical and experimental stress–strain curves for an enclosing pressure of 6 MPa.

**Figure 8:**
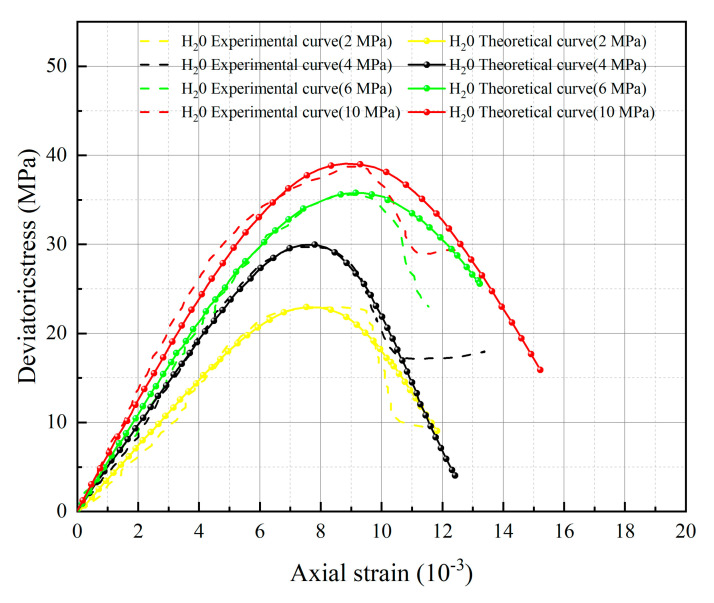
Comparison of theoretical and experimental stress–strain curves for water action.

Figure 5, Figure 6, Figure 7 and Figure 8 below present a comparison between the experimental and model curves of rock under triaxial stress, utilizing the chemical damage variables, water damage variables, and constitutive model parameters that were previously obtained.

Compare the experimental curves under different hydrochemistry corrosion and different confining pressures with the constitutive model’s theoretical curve, as shown in Figure 5, Figure 6, Figure 7 and Figure 8. The results show that the model’s theoretical curves and experimental curves are appropriately in agreement, indicating that the established rock chemical corrosion-water-confining pressure coupled damage constitutive model can be consistent in the stress–strain characteristics of the rock triaxial compression process.

The analysis of 13 groups of data showed that there is a certain rule in the parameters *m* and *F*_0_ of the damage constitutive model based on the SMP strength criterion. Many scholars [41,42,43] believe that parameter *m* may be related to the strength of rock microelements, while parameter *F*_0_ represents the macroscopic average strength of rock. The parameters *m* and *F*_0_ under the SMP criterion are still closely linked to the strength of rock microelements and the macroscopic average strength of rock. Figure 5, Figure 6, Figure 7 and Figure 8 show that the stress peak of the curve increases with the increase of *F*_0_ and *m*. However, the change of *F*_0_ and *m* has no effect on the linear deformation curve before the peak value. The effect of *F*_0_ and *m* on the nonlinear deformation part of the stress–strain curve of rock, especially the curve after the peak value, is significant and can change the shape of the curve.

**Figure 9:**
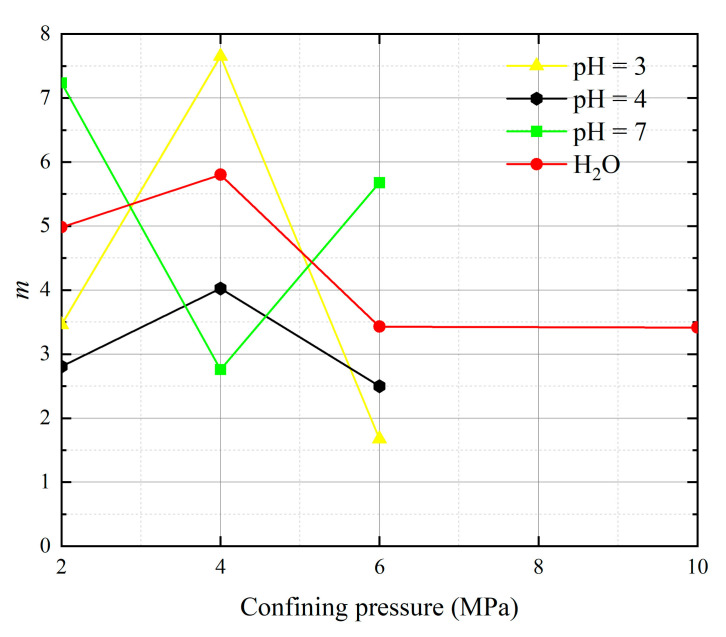
Effects of different values of *pH* and confining pressures on model parameter *m*.

**Figure 10:**
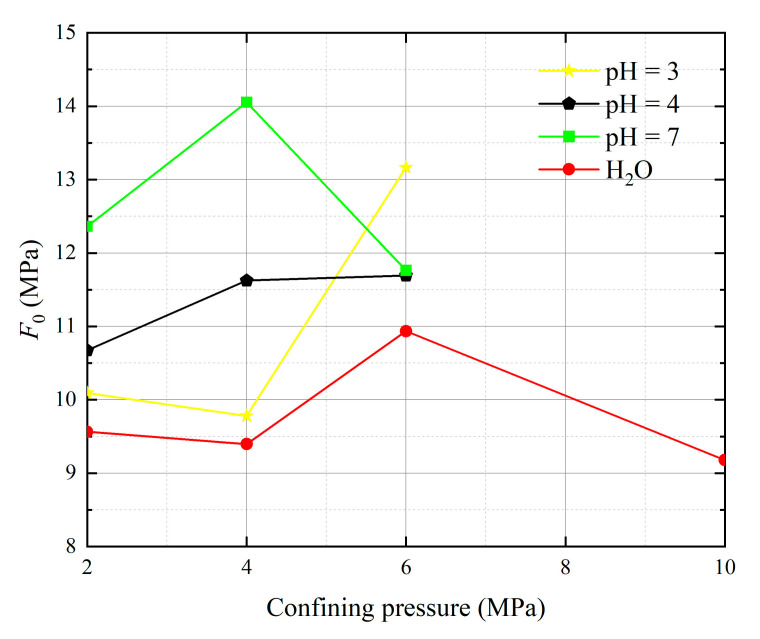
Effects of different values of *pH* and confining pressures on model parameter *F*_0_.

Figure 9 and Figure 10 illustrate the two Weibull distribution parameters (*m* and *F*_0_) trends with changes in different values of *pH* and confining pressures. As can be seen in Figure 9, at constant confining pressure, the parameter *m* shows an overall increasing trend with decreasing *pH* value. This means that the larger the value of *m*, the greater the degree of corrosion difference of the rock body in the region. With increasing confining pressure, the parameter *m* initially increases and then decreases, with a turning point appearing at the confining pressure of 4 MPa, showing a non-linear relationship with confining pressure. The parameter *m* essentially ceases to decrease during the course of the confining pressure from 6 MPa to 10 MPa. At *pH* = 7, a special case occurs where the parameter *m* decreases and then increases, *m* reflects the degree of dispersion of the data, which is caused by many factors such as measurement errors, errors due to fluctuating conditions, and regional differences in rock corrosion. In Figure 10, *F*_0_ represents the peak of the curve (maximum probability) when the corrosivity is related to the mean value of all corrosion samples but not equal to the sample mean. With increasing confining pressure, at *pH* = 3 and water, the parameter *F*_0_ initially decreases and then increases, and the turning point occurs at a confining pressure of 4 MPa. At *pH* = 4 and *pH* = 7, the parameter *F*_0_ increases and then decreases, and the turning point also occurs at 4 MPa. The parameter *F*_0_ continues to drop more sharply during the course of the confining pressure from 6 MPa to 10 MPa. When *m* is unchanged, as *F*_0_ increases rock peak strength increases, *F*_0_ reflects the size of macro average strength of rock; when fixed *F*_0_ is unchanged, *m* indicates the concentration degree of rock micro element strength distribution, and its physical significance reflects the degree of nonuniformity of rock acid corrosion.

By observing Figure 5, Figure 6, Figure 7 and Figure 8, it becomes apparent that the peak stress of the rock gradually diminishes as the *pH* value decreases under identical confining pressure conditions. Conversely, when subjected to the same acid corrosion, the peak stress of the rock increases with higher levels of confining pressure. This corresponds with Wang’s experimental results, indicating that the rock damage constitutive model based on Weibull distribution and SMP criterion constructed in this study can accurately reflect the changes in rock strength under different *pH* corrosion and confining pressures, thereby demonstrating the feasibility of defining chemical damage factors considering *pH* value and time. Defining water damage variables based on the moles of soluble cementitious material was lost in the reaction with water instead of macroscopic damage variables.

## 6. Conclusions

The present article centers on rocks that are susceptible to chemical erosion and aqueous deterioration. The present study analyzed available experimental data, which revealed that the corruption of rock mechanical properties due to chemical corrosion and water damage can be attributed to the loss of soluble cementitious material present in the rocks. The article provided a quantification of both the chemical and water damage incurred by the rocks. A constitutive model was established for the chemical corrosion-water-confining pressure coupling damage in rocks based on damage theory. The study focused on the damage evolution characteristics of the rocks. The following deductions were made.

(1) The spatial mobilized plane (SMP) criterion considering axial stress is introduced, the total damage variable *D*_S_ considering chemical damage, water damage and mechanical damage is deduced, and the rock damage constitutive model considering chemical-mechanics-hydro (C-M-H) coupled damage is established, which can reflect the stress–strain characteristics in the process of rock triaxial compression: With the increase of confining pressure, the peak stress and strain of rock under the same conditions increase; chemical damage and water damage will lead to the decrease of rock strength, and the degree of decrease will increase with the decrease of *pH* value.
(2) Stone is selected as the sample for acid corrosion treatment at *pH* 3, 4, and 7, and a chemical damage factor is defined that coupled the *pH* value and duration of exposure. The proposed damage constitutive model employs the extremum method to ascertain the two Weibull distribution parameters (*m* and *F*_0_) by theoretical derivation and exhibits satisfactory conformity between the theoretical and experimental curves. The damage constitutive model can be consistent in the stress–strain characteristics of the rock triaxial compression process, which verifies the rationality and reliability of the model parameters.
(3) The parameters *m* and *F*_0_ under the SMP criterion are still closely linked to the strength of rock microelements and the macroscopic average strength of rock. The analysis of 13 groups of comparative data shows the stress peak of the curve increases with the increase of damage model parameters *F*_0_ and *m*. At constant confining pressure, the parameter *m* shows an overall increasing trend with decreasing *pH* value. The larger the value of *m*, the greater the degree of corrosion difference of the rock body in the region. When *m* is unchanged, as *F*_0_ increases, rock peak strength increases.
(4) In terms of the validation of the rock damage constitutive model considering chemical-mechanics-hydro (C-M-H) coupled damage, this study adopts the experimental data from the current literature to validate the chemical damage, water damage, and force damage separately, the verification method is not a true coupling. In the future, we will conduct some experiments on rocks under the coupling of water chemistry and confining pressure to further validate this damage constitutive model.
(5) When considering the heterogeneity of the rock itself, the damage constitutive model established in this paper has no specific rock type parameters, and the required model parameters (*m* and *F*_0_) can be obtained through routine triaxial tests in the laboratory. The modified damage constitutive model can not only be applied to the chemical-mechanics-hydro (C-M-H) coupled damaged sandstone, but also can well describe the degree of damage and strength characteristics in the pre-peak stage under triaxial compression. It has certain theoretical significance for mining and railway construction that traverses the areas affected by acid rain and an intense rainfall climate.

## Figures and Tables

**Table 1:** Chemical composition of sandstone %.

Project	SiO_2_	Fe_2_O_3_	Al_2_O_3_	CaO	MgO	TiO_2_	K_2_O	Na_2_O
Maximum value	55.0	6.3	13.0	10.2	6.3	1.0	1.8	2.1
Minimum value	46.0	2.5	9.0	3.4	1.3	0.3	1.6	1.4
Average value	51.2	4.6	11.3	7.0	3.5	0.6	1.7	1.7

**Table 2:** Calculation table for chemical damage of sandstone in *pH* 3, 4 and water environment.

Substance	MgO	Fe_2_O_3_	Al_2_O_3_	CaO	K_2_O	Na_2_O	Total
Weight (g)	17.50	23.00	56.50	35.00	8.50	8.50	
Molar mass	40	160	102	56	94	62	
Moles (mol)	0.4375	0.1438	0.5539	0.6250	0.0904	0.1370	1.9876
Proportion (%)	22.01	7.23	27.87	31.44	4.55	6.89	
*pH* = 3. Molar number of damaged soluble solids	1.10 × 10^−4^	1.20 × 10^−5^	4.65 × 10^−5^	1.57 × 10^−4^	2.28 × 10^−5^	3.45 × 10^−5^	3.8280 × 10^−4^
*pH* = 4. Molar number of damaged soluble solids	1.08 × 10^−5^	1.16 × 10^−6^	4.57 × 10^−6^	1.49 × 10^−5^	2.23 × 10^−6^	3.44 × 10^−6^	3.8032 × 10^−5^

**Table 3:** Parameter table of sandstone model calculation.

Operating Environment	Confining Pressure **(**MPa**)**	***ԑ*_p_ (**10^−3^)	***σ*_p_** (MPa**)**	*m*	***F*_0_** (MPa)
*pH* = 3	2	17.196	13.564	3.460	10.094
	4	11.951	24.482	7.651	9.780
	6	16.963	26.191	1.677	13.165
*pH* = 4	2	15.219	14.587	2.807	10.674
	4	11.964	25.858	4.026	11.625
	6	13.954	27.733	2.499	11.694
*pH* = 7	2	8.413	23.209	7.237	12.363
	4	12.390	26.933	2.759	14.056
	6	11.761	37.122	5.678	11.763
H_2_O	2	7.675	22.854	4.983	9.564
	4	7.848	29.957	5.802	9.397
	6	9.419	35.857	3.429	10.936
	10	9.134	38.797	3.415	9.178

## Data Availability

Data will be made available on request.
